# Biologic Therapies: A Systematic Review of the Indications, Efficacy, Safety, and Outcomes in Ear, Nose, and Throat Diseases

**DOI:** 10.7759/cureus.101059

**Published:** 2026-01-07

**Authors:** Zainab Al Saloom, Mahmood Alawainati, Zahraa Abdeen, Israa Qadmi, Alaa Mandeel, Khaled AlAani

**Affiliations:** 1 Ear, Nose, and Throat (ENT), Government Hospital Bahrain, Manama, BHR; 2 Medicine, Royal College of Surgeons in Ireland, Manama, BHR; 3 Family Medicine, Primary Healthcare Centers, Manama, BHR; 4 Ear, Nose, and Throat (ENT), Alsalam Hospital, Manama, BHR; 5 Ear, Nose, and Throat (ENT), Salmaniya Medical Complex, Manama, BHR; 6 Surgery, College of Medicine and Medical Sciences, Arabian Gulf University, Manama, BHR

**Keywords:** biological therapy, labyrinth diseases, nasal polyps, otolaryngology, rhinosinusitis

## Abstract

Biologic therapies, such as omalizumab, mepolizumab, and dupilumab, are novel therapeutic agents that offer a targeted approach for managing chronic inflammatory and immune-mediated ear, nose, and throat (ENT) conditions. However, the indications, efficacy, and adverse events of these medications in various ENT disorders have not been studied before. Therefore, this systematic review was performed to evaluate their indications, clinical efficacy, and safety in ENT management. A systematic search was conducted in accordance with the Preferred Reporting Items for Systematic Reviews and Meta-Analyses (PRISMA) guidelines across five major databases: PubMed, Embase, Cochrane Library, Medline, and Web of Science. Studies published between 2015 and May 2025 were considered, and additional manual searches and reference screening were also performed. Eligible studies included those written in English, assessing biologics in ENT diseases, and reporting on at least one of the following outcomes: clinical efficacy, safety, or indications. The study characteristics, population details, medications used, and outcomes were assessed. Study quality was assessed using the Joanna Briggs Institute critical appraisal tool, with disagreements resolved by a senior assessor. A total of 497 records were identified, and after screening and quality assessment, 10 trials involving 2,406 patients were included. The primary indication of biologics use across nine studies was chronic rhinosinusitis with nasal polyps (CRSwNP), while one study assessed allergic rhinitis. Most trials compared biologics to a placebo, except one study that evaluated mepolizumab in combination with functional endoscopic sinus surgery. Biologics consistently demonstrated clinically significant reductions in endoscopic nasal polyp scores (P<0.05) and improvements in Visual Analogue Scale (P<0.05), SinoNasal Outcome Test (P<0.05), and olfactory function (P<0.001). Imaging findings (Lund-Mackay scores) were also in favor of biologics (P<0.05). Furthermore, biologics were associated with reduced need for repeat surgery (mepolizumab), lower serum immunoglobulin E (IgE) levels (dupilumab), and better productivity and quality of life (omalizumab). The safety profile was favorable, with nasopharyngitis (4-47%), headache (7-25%), and injection-site reactions (6-40%) being the most common adverse events, while no fatal events were reported (0%). In summary, biologics improved clinical symptoms, nasal polyp scores, olfaction, and quality of life in ENT conditions, particularly CRSwNP and allergic rhinitis, with favorable safety profiles and additional benefits, including reduced need for surgery and lower IgE levels.

## Introduction and background

Biologic therapies, a diverse group of drugs that act directly on the immune system, have revolutionized the management of several chronic inflammatory and immune-mediated diseases across various medical fields, including otorhinolaryngology [1]. By targeting specific molecular pathways, these agents have provided safe and effective alternatives to conventional therapies, such as antihistamines, corticosteroids, and surgical interventions [2]. Biologic agents such as omalizumab, mepolizumab, and dupilumab, as well as other agents, are effective in treating ear, nose, and throat (ENT) conditions by modulating the immune response through various mechanisms, including monoclonal antibodies, cytokine inhibitors, receptor antagonists, and fusion proteins [3].

The biologics target different inflammatory pathways, which explains their varied efficacy and indications. For instance, dupilumab acts by blocking the functions of interleukin-4 (IL-4) and IL-13, leading to improvements in nasal polyp size, olfaction, and IgE levels, making dupilumab particularly effective in patients with chronic rhinosinusitis with nasal polyps (CRSwNP) [4]. Mepolizumab targets IL-5, an essential cytokine for eosinophil differentiation and survival. Therefore, it modulates eosinophilic inflammation and is particularly effective in CRSwNP, particularly in patients with high tissue eosinophilia [5]. Omalizumab binds to IgE, preventing its interaction with receptors on mast cells and basophils. This mechanism explains its efficacy not only in CRSwNP but also in allergic rhinitis, where IgE-mediated hypersensitivity plays a dominant role [6].

The targeted mechanisms of biologic agents confer substantial benefits for patients with severe, treatment-resistant conditions, including CRSwNP, allergic rhinitis, and autoimmune ear diseases [7,8]. As their uses broaden, the focus has shifted toward precision medicine to ensure that biologics are prescribed to patients who will benefit most, balancing efficacy with cost-effectiveness [7-9].

Despite promising results, biologics are not considered first-line treatments and are reserved for cases where conventional therapies are ineffective or contraindicated. EPOS/EUFOREA 2023 provides clear clinical guidelines for biologic eligibility based on inflammatory markers, symptom severity, and comorbid conditions like asthma. However, the widespread use of these agents remains limited due to their high costs [9].

Although biologics are becoming integral in managing several ENT diseases, no previous systematic reviews have comprehensively synthesized the clinical indications, efficacy, and safety profiles of biologics in the management of ENT conditions. Therefore, this systematic review aims to determine the indications, clinical efficacy, and safety of biologic therapies in the treatment of different ENT conditions.

## Review

Design

The present systematic review was conducted in 2025 in accordance with the Preferred Reporting Items for Systematic Reviews and Meta-Analyses (PRISMA) guidelines. It included studies published between May 2015 and May 2025.

Search strategy

A comprehensive search was done across several electronic databases, including PubMed, Embase, Cochrane Library, Medline, and Web of Science. The search strategy combined two strings; the first one was related to biologic therapies (“Omalizumab”, “Mepolizumab”, “Dupilumab”, “biologic agents”, “monoclonal antibodies”, and “cytokine inhibitors”), and the second string was related to ENT conditions (including “chronic rhinosinusitis with nasal polyps”, “CRSwNP”, “allergic rhinitis”, “autoimmune inner ear disease”, “ENT”, “Otolaryngology”, and “ENT biologics”). The search involved at least one word from each string. Furthermore, additional manual searches and screening of the reference lists of relevant studies were conducted to ensure that all eligible studies were assessed.

All search results were exported to an MS Excel (Microsoft Corporation, Redmond, Washington) sheet and organized by title, author, and journal. Duplicate records were removed, and the remaining studies were screened based on titles and abstracts. Full texts of potentially relevant articles were then assessed for eligibility. Studies were included if they were published in English, investigated biologic therapies in ENT conditions, and reported on at least one of the following outcomes: clinical efficacy, safety profile, or indications. Exclusion criteria included non-English articles, case reports, reviews, conference abstracts, and studies lacking full-text access or relevant outcomes.

The following details were taken from the included studies: year of publication, intervention, comparison, indication, follow-up duration, sample size, Joanna Briggs Institute (JBI) results, and outcomes.

Quality assessment tool

The quality of the included studies was independently assessed by two reviewers using the JBI critical appraisal tool [10]. Only studies meeting predefined quality and relevance criteria were included. The tool assessed randomization, allocation concealment, baseline similarities, blinding, identical treatment except for intervention, follow-up completeness, intention-to-treat analysis, consistent outcome measurement, reliable outcome measurement, and appropriate design and analysis [10]. In cases of disagreement between reviewers, a senior author served as a third assessor, and their evaluation determined the final inclusion status.

Analysis

Descriptive analysis using numbers and frequencies was performed. A meta-analysis was not conducted due to heterogeneity of data, including different tools and follow-up durations. The final pool of studies was analyzed to summarize the effects of biologic therapies on symptom control, polyp reduction, hearing outcomes, quality of life, and safety profiles [10]. Two reviewers independently extracted the data. Any inconsistencies were resolved through discussion and agreement.

Results

**Figure 1:**
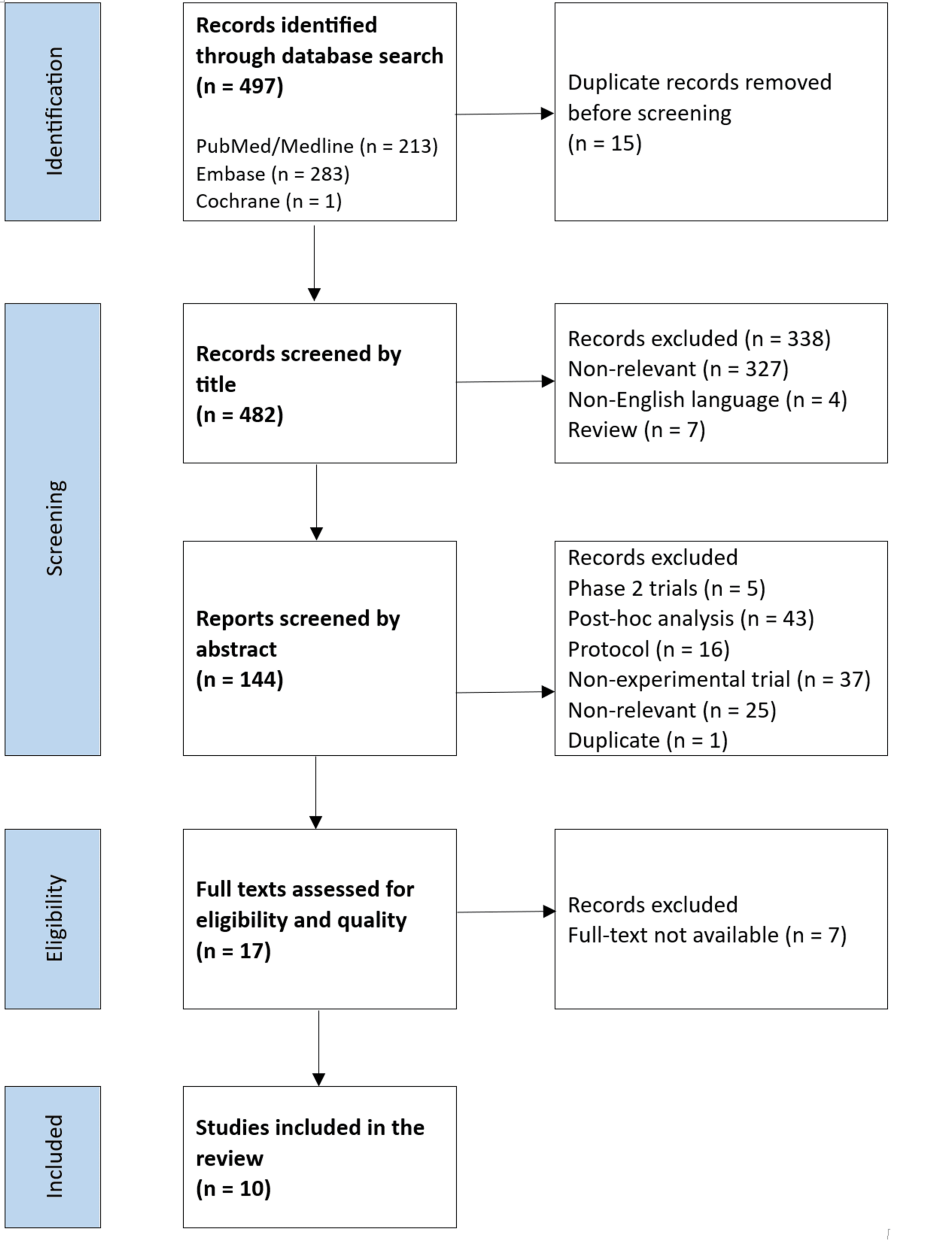
PRISMA Flowchart for the Study Selection PRISMA: Preferred Reporting Items for Systematic Reviews and Meta-Analyses

A total of 497 records were retrieved through database searches (PubMed/Medline = 213, Embase = 283, Cochrane = 1). After removing 15 duplicates, 482 records were screened by title, resulting in the exclusion of 338 records (non-relevant = 327, non-English = 4, reviews = 7). The remaining 144 records underwent abstract screening, from which 127 were excluded (phase 2 clinical trials = 5, post hoc analyses = 43, protocols = 16, non-experimental studies = 37, non-relevant studies = 25, and duplicates = 1). Then, 17 full-text articles underwent eligibility and quality assessment. Seven studies were excluded due to poor quality assessment, leaving 10 studies that met the inclusion criteria and were included in the final systematic review analysis. The study selection process is illustrated in Figure 1.

As shown in Table 1, a total of 10 randomized controlled trials and 2406 patients were included in this systematic review [11-20]. Of these, nine studies assessed interventions for CRSwNP [11-18,20], while one study focused on allergic rhinitis cases [19]. Three biologic agents were investigated: dupilumab (n = 3) [11,13,20], mepolizumab (n = 4) [12,14,17,18], and omalizumab (n = 3) [15,16,19]. The sample sizes ranged from 30 to 724 participants.

**Table 1:** Characteristics of The Included Randomized Controlled Trials CRSwNP: Chronic rhinosinusitis with nasal polyps; FESS: Functional endoscopic sinus surgery

Number	Study, Year	Sample	Intervention Group	Comparison Group	Assessed Condition	Follow-Up (Weeks)
1	Bachert et al., 2016 [11]	60	Dupilumab (n=30)	Placebo (n=30)	CRSwNP	16
2	Bachert et al., 2017 [12]	107	Mepolizumab (n=54)	Placebo (n=53)	CRSwNP	25
3	Bachert et al., 2019 [13]	724	Dupilumab (n=438)	Placebo (n=286)	CRSwNP	52
4	Fujieda et al., 2024 [14]	169	Mepolizumab (n=84)	Placebo (n=85)	CRSwNP	52
5	Gevaert et al., 2022 [15]	249	Omalizumab (n=124)	Placebo (n=125)	CRSwNP	52
6	Gevaert et al., 2020 [16]	265	Omalizumab (n=134)	Placebo (n=131)	CRSwNP	24
7	Han et al., 2021 [17]	407	Mepolizumab (n=206)	Placebo (n=201)	CRSwNP	52
8	Homøe et al., 2025 [18]	58	FESS + Mepolizumab (n=29)	FESS alone (n=29)	CRSwNP	26
9	Okubo et al., 2020 [19]	337	Omalizumab (n=162)	Placebo (n=175)	Allergic Rhinitis	12
10	Pelletier et al., 2024 [20]	30	Dupilumab (n=16)	Placebo (n=14)	CRSwNP	52

Table 2 presents the quality assessment findings of the included studies. The included randomized controlled studies were of high methodological quality, with consistent use of randomization, intention-to-treat analysis, and appropriate statistical methods across all studies. Blinding was generally well implemented, although a few trials reported unclear allocation concealment or assessor blinding.

**Table 2:** Quality Assessment of the Included Randomized Controlled Trials

Study, Year	Randomization	Allocation Concealment	Baseline Similarity	Participant Blinding	Provider Blinding	Assessor Blinding	Identical Treatment (Except Intervention)	Follow-Up Completeness/Handling	Intention-to-Treat Analysis	Consistent Outcome Measurement	Reliable Outcome Measurement	Appropriate Statistics	Appropriate Design
Bachert et al., 2016 [11]	Yes	Yes	Yes	Yes	Yes	Yes	Yes	Yes	Yes	Yes	Yes	Yes	Yes
Bachert et al., 2017 [12]	Yes	Yes	Yes	Yes	Yes	Yes	Yes	Yes	Yes	Yes	Yes	Yes	Yes
Bachert et al., 2019 [13]	Yes	Yes	Yes	Yes	Yes	Yes	Yes	Yes	Yes	Yes	Yes	Yes	Yes
Fujieda et al., 2024 [14]	Yes	Yes	Yes	Yes	Yes	Unclear	Yes	Yes	Yes	Yes	Yes	Yes	Yes
Gevaert et al., 2022 [15]	Yes	Yes	Yes	Yes	Yes	Yes	Yes	Yes	Yes	Yes	Yes	Yes	Yes
Gevaert et al., 2020 [16]	Yes	Yes	Yes	Yes	Yes	Yes	Yes	Yes	Yes	Yes	Yes	Yes	Yes
Han et al., 2021 [17]	Yes	Yes	Yes	Yes	Yes	Yes	Yes	Yes	Yes	Yes	Yes	Yes	Yes
Homøe et al., 2025 [18]	Yes	Unclear	Yes	No	No	Yes	Yes	Yes	Yes	Yes	Yes	Yes	Yes
Okubo et al., 2020 [19]	Yes	Yes	Yes	Yes	Yes	Yes	Yes	Yes	Yes	Yes	Yes	Yes	Yes
Pelletier et al., 2024 [20]	Yes	Unclear	Yes	Yes	Yes	Unclear	Yes	Yes	Yes	Yes	Yes	Yes	Yes

The comparison group in nine out of 10 studies was a placebo, while one study [18] compared combination therapy (FESS (functional endoscopic sinus surgery) + mepolizumab) against FESS alone [18]. In addition, the follow-up periods ranged from 12 weeks to 52 weeks. The quality assessment using the JBI critical appraisal tool revealed that seven studies achieved a score of 11/13, while three studies scored between 8 and 10, indicating high quality levels [11-20].

Nine studies assessed nasal polyps using the Endoscopic Nasal Polyp Score (NPS) and showed that biologics (dupilumab, mepolizumab, and omalizumab) produced significantly greater bilateral NPS reductions versus placebo. Four studies evaluated polyps with Lund-Mackay CT (LMK-CT) scores, reporting significant reductions with dupilumab and mepolizumab, although one found similar CT improvements between placebo and dupilumab [9].

Symptomatic improvement was measured using the Visual Analogue Scale (VAS) or similar tools, and all studies reported significant VAS and nasal symptom relief with biologics versus placebo. Sinonasal outcomes assessed by SNOT (SinoNasal Outcome Test) scores showed significant SNOT-22 improvements in 9/10 studies, with greater quality-of-life gains for dupilumab, mepolizumab, and omalizumab compared to placebo. Smell function (UPSIT (University of Pennsylvania Smell Identification Test), VAS smell) improved significantly in biologic-treated groups; dupilumab produced marked UPSIT gains [11,13,20], and mepolizumab also improved smell (P<0.001; P=0.020) [12,17], though one study reported non-significant UPSIT results (P = 0.30) [17]. Additional findings included reduced serum IgE with dupilumab [11], lower surgical intervention rates with mepolizumab (P = 0.006) [12], and improved quality of life and work productivity with omalizumab (P < 0.001) (Table 3) [16].

**Table 3:** Summary of the Main Findings of the Studies Included in the Review NPS: Endoscopic nasal polyp score (0–8; higher = worse); VAS: Visual Analogue Scale (0–10 cm; higher = worse symptoms); LMK-CT: Lund-Mackay CT score (0–24; higher = worse); SNOT: SinoNasal Outcome Test-22 (0–110; higher = worse QoL); UPSIT: University of Pennsylvania Smell Identification Test (0–40; higher = better); NA: Not Assessed

Study, Year	Endoscopic Nasal Polyp Score (NPS)	Visual Analogue Scale (VAS)	Imaging Score – LMK-CT	Sinonasal Score – SNOT	Smell Score – UPSIT	Other Outcomes
Bachert et al., 2016 [11]	Dupilumab resulted in a greater reduction in bilateral NPS compared to placebo (P<0.001)	Better improvements in sinusitis symptoms vs. placebo using VAS (P=0.008)	Higher reduction in LMK-CT total score in the dupilumab group vs. placebo (P<0.001)	SNOT scores significantly lower post-treatment with dupilumab vs. placebo (P<0.001)	Significant improvement in smell in the dupilumab group vs. placebo (P<0.001)	Significant reduction in IgE in the dupilumab group vs. placebo (P<0.001)
Bachert et al., 2017 [12]	Reduction of NPS was higher in mepolizumab vs. placebo (P=0.025)	Significant improvement in VAS vs. placebo (P=0.001)	NA	Significantly greater improvements in SNOT-22 vs. placebo	UPSIT: NA; VAS scores showed better smell function in mepolizumab vs. placebo (P<0.001)	A higher proportion of Mepolizumab patients no longer required surgery at week 25 (P=0.006)
Bachert et al., 2019 [13]	Significant difference in NPS favoring dupilumab vs. placebo (P<0.0001)	Nasal congestion scores decreased significantly vs. placebo (P<0.0001)	Better changes in LMK-CT vs. placebo (P<0.001)	SNOT scores significantly improved with Dupilumab vs. placebo	Anosmia decreased; UPSIT improved vs. placebo (P<0.0001)	NA
Fujieda et al., 2024 [14]	Significant reduction in ENPS at Week 52 with mepolizumab vs. placebo (P=0.043)	Significant reduction in overall VAS score (P=0.003)	Greater reduction in LMK-CT vs. placebo (P=0.01)	SNOT-22 total score reduced more in Mepolizumab vs. placebo (P=0.01)	Loss of smell by VAS improved in favor of Mepolizumab (P=0.009)	NA
Gevaert et al., 2022 [15]	Significant difference in NPS with Omalizumab (P<0.0001)	Lower rates of postnasal drip, runny nose, sense of smell vs. placebo	NA	SNOT-22 improved in omalizumab vs. placebo (P<0.0009)	UPSIT scores higher in the omalizumab group	Nasal congestion improved more with omalizumab vs. placebo (P<0.0001)
Gevaert et al., 2020 [16]	Significant improvement in mean NPS with omalizumab vs. placebo (P<0.0001)	Symptoms like smell (P=0.0161), postnasal drip (P=0.0001), runny nose (P<0.0023) improved vs. placebo	NA	Significant improvement in SNOT score (P<0.0001)	UPSIT improved vs. placebo (P<0.0024)	NA
Han et al., 2021 [17]	NPS improved from baseline with mepolizumab vs. placebo (P<0.0001)	VAS significantly improved (P<0.0001)	NA	SNOT-22 reduced more in mepolizumab vs. placebo (P=0.0032)	UPSIT changes not significant (P=0.30), VAS improvement significant (P=0.020)	NA
Homøe et al., 2025 [18]	NPS improved significantly with Mepolizumab regardless of FESS (P<0.001)	VAS reduced significantly with Mepolizumab (P<0.001); FESS did not influence outcomes	NA	Large polyps improved (P=0.002); small polyps showed no difference (P=0.862)	NA	All patients improved after 6 months (P<0.05)
Okubo et al., 2020 [19]	NA	Significant differences in mean nasal symptom scores favoring Omalizumab (P<0.001)	NA	NA	NA	Omalizumab significantly improved QoL and work productivity (P<0.001)
Pelletier et al., 2024 [20]	Dupilumab significantly reduced NPS vs. placebo (P<0.001)	Significant improvement in VAS vs. placebo (P<0.05)	CT scan scores showed comparable improvement (P<0.0001)	SNOT scores improved significantly vs. placebo (P<0.05)	UPSIT showed greater improvement in the dupilumab group (P<0.05)	NA

As shown in Table 4, nasopharyngitis (4%-47%), headache (7%-25%), and injection-site reaction (6%-40%) were the most frequently reported adverse events across studies. In general, comparable rates of side effects were observed between the placebo and biologic treatment groups. None of the studies reported any fatal adverse events related to the intervention.

**Table 4:** Adverse Events of Biologics Used in ENT NA: Not Available

Study (Year)	Intervention	% Adverse Events (Placebo)	% Adverse Events (Intervention)	Common Adverse Events in the Intervention Group	Fatal Adverse Events (%)
Bachert et al., 2016 [11]	Dupilumab	83.3%	100%	Nasopharyngitis (47%), Injection-site reactions (40%), Headache (20%)	0%
Bachert et al., 2017 [12]	Mepolizumab	81%	75%	Headache (25%), Nasopharyngitis (19%), Oropharyngeal pain (11%)	0%
Bachert et al., 2019 [13]	Dupilumab	74%	69%	Nasopharyngitis (13%), Headache (7%), Epistaxis (6%), Injection-site erythema (6%)	0%
Fujieda et al., 2024 [14]	Mepolizumab	76%	81%	COVID-19 (18%), Nasopharyngitis (11%), Headache (7%)	0%
Gevaert et al., 2022 [15]	Omalizumab	49.6%	43.5%	Nasopharyngitis (4%)	0%
Gevaert et al., 2020 [16]	Omalizumab	58.5%	50.4%	Headache (8.1%), Nasopharyngitis (5.9%), Injection-site reaction (5.2%)	0%
Han et al., 2021 [17]	Mepolizumab	9%	15%	Nasopharyngitis (25%), Headache (18%), Epistaxis (8%), Oropharyngeal pain (8%)	0%
Homøe et al., 2025 [18]	Mepolizumab	NA	NA	NA	NA
Okubo et al., 2020 [19]	Omalizumab	27.4%	27.3%	Nasopharyngitis (9.3%), Pharyngitis (4.3%), Influenza (2.5%)	0%
Pelletier et al., 2024 [20]	Dupilumab	NA	NA	No infections	0%

Discussion

This study aimed to determine the indications, safety, and efficacy of biologic therapies in the management of ENT conditions. The results showed that biologics, including dupilumab, mepolizumab, and omalizumab, improved NPS, sinonasal symptoms, olfactory function, and quality of life in patients with CRSwNP and allergic rhinitis, with favorable safety profiles. Additionally, these agents were associated with a reduced need for repeat surgical interventions and with immunological improvements, such as lower serum IgE levels.

The reviewed studies consistently revealed that biologics, including dupilumab, mepolizumab, and omalizumab, significantly improved sinonasal outcomes in patients with CRSwNP compared to placebo, as evidenced by VAS scores. This reflects that these medications reduce nasal congestion and overall symptom burden [11-14,17,18,20]. This is further supported by the significant reductions in SNOT-22 scores, which were reported in all studies that assessed this outcome [11,12,15,16].

Furthermore, most biologics demonstrated marked efficacy in relation to olfactory function as noted by the improvements in UPSIT scores and reductions in loss-of-smell symptoms [11,12,15,16,20]. Although mepolizumab showed favorable improvements in NPS and smell-related VAS scores, UPSIT improvements were less consistent, with some studies not reporting statistically significant differences [15-17]. Although mepolizumab consistently improved VAS scores and SNOT-22 outcomes, smell-related UPSIT improvements were less evident, with some studies showing no statistically significant differences [17]. Furthermore, biologics reduced the need for surgical interventions and led to improvements in immunological markers such as serum IgE [11-12] and better quality of life and work productivity scores [19].

Objective assessment by endoscopic and radiologic scoring confirmed the efficacy of biologics in CRSwNP. Significant reductions in NPS were consistently reported with dupilumab [11,13,20], mepolizumab [12,14,17,18], and omalizumab [15,16]. Similarly, LMK-CT scores improved significantly with dupilumab [11,13] and mepolizumab [14]. Notably, Pelletier et al. (2024) observed CT improvements in both the dupilumab and placebo arms, though earlier studies showed greater benefit with biologics [20]. These findings show that biologics provide not only symptomatic improvements but also measurable structural and radiologic improvements in various ENT disorders [20].

Recent data published in 2025 further reinforce and extend our findings. Real-world evidence confirms that the efficacy of biologics observed in RCTs is reproducible in routine practice, with Cai et al.’s meta-analysis showing consistent improvements in NPS, SNOT-22, and smell scales across nearly 4,000 patients [21]. Importantly, Book et al. demonstrated that dupilumab maintains durable effects beyond three years, with the added practical advantage of safely lengthening injection intervals - a finding with significant implications for patient adherence and healthcare resource allocation. Notably, the study allowed extension of injection intervals (six weeks), with no loss of efficacy - a very practical finding for maintenance strategies [22]. Xu et al.’s review of 29 studies highlighted the heterogeneity in response criteria across studies with pooled moderate-to-excellent response rates ranging from ~39-88% (comprehensive criteria) to ~63-83% (single-measure criteria) [23].

The safety profile observed across the included studies was favorable, with adverse events such as nasopharyngitis, headache, and injection-site reactions generally mild and comparable to those in placebo groups. Importantly, no treatment-related fatal events were reported. This emphasizes the overall tolerability and safety of biologics in ENT practice.

In terms of indications, dupilumab and mepolizumab have primarily been used in patients with CRSwNP, whereas omalizumab has been used in allergic rhinitis. Beyond these approved indications, biologic therapies have also been tested for the treatment of other conditions, including various ENT autoimmune diseases; however, their efficacy remains uncertain and remains under investigation [7,9].

Clinically, physicians should consider the use of biologic therapies in patients with refractory CRSwNP, severe eosinophilic asthma, or allergic rhinitis who have failed first-line and second-line treatments, particularly when there is evidence of type 2 inflammation as indicated by elevated eosinophils, serum IgE, or comorbid atopic conditions. Careful patient selection based on these biomarkers, disease severity, and comorbidities can enhance therapeutic outcomes, reduce the need for repeated surgical interventions, and improve overall quality of life.

This systematic review has several strengths, including adherence to PRISMA guidelines, high quality levels of included studies, and the synthesis of outcomes across multiple validated clinical scores. However, it has some limitations as well. Most studies had relatively short follow-up periods and heterogeneity of study design (different endpoints and scoring tools), making it difficult to conclude the long-term efficacy, safety, and durability of biologic responses. In addition, meta-analysis and economic analysis were not reported due to the heterogeneity of the studies.

## Conclusions

In summary, dupilumab, mepolizumab, and omalizumab were efficacious in improving sinonasal symptoms, reducing nasal polyp burden, enhancing smell function, and improving quality of life in patients with CRSwNP and in cases of allergic rhinitis. These agents demonstrated a favorable safety profile, with no major safety concerns reported. Despite promising results, long-term data on the durability of response and on the comparative effectiveness of different biologic agents in ENT diseases remain limited. Therefore, large-scale and head-to-head trials are needed to form evidence-based treatment algorithms.
